# Barriers and opportunities for community engagement in UAV-based dengue management in rural Malaysia

**DOI:** 10.1371/journal.pone.0322321

**Published:** 2025-04-30

**Authors:** Nazri Che Dom, Rahmat Dapari, Muhamad Shahrizal Shapien, Qamarul Nazri Harun, Siti Aekbal Salleh, Ahmad Falah Aljaafre

**Affiliations:** 1 Centre of Environmental Health & Safety, Faculty of Health Sciences, Universiti Teknologi MARA (UiTM), UITM Cawangan Selangor, Puncak Alam, Malaysia; 2 Integrated Mosquito Research Group (I-MeRGe), Universiti Teknologi MARA (UiTM), UITM Cawangan Selangor, Puncak Alam, Malaysia; 3 Institute for Biodiversity and Sustainable Development (IBSD), Universiti Teknologi MARA, Shah Alam, Malaysia; 4 Integrated Dengue Research and Development, Faculty of Medicine and Health Sciences, Universiti Putra Malaysia, Serdang, Malaysia; 5 Department of Community Health, Faculty of Medicine and Health Sciences, Universiti Putra Malaysia, Serdang, Malaysia; 6 School of Information Science, College of Computing, Informatics and Mathematics, Universiti Teknologi MARA, Shah Alam, Malaysia; 7 Department of Communication and Computer Engineering, Tafila Technical University, Tafila, Jordan; Universiti Putra Malaysia, MALAYSIA

## Abstract

Dengue fever remains a significant public health issue in Malaysia, particularly in rural areas where unique challenges such as dispersed populations, limited infrastructure, and distinct socio-cultural dynamics complicate vector control efforts. Drone technology has emerged as an innovative tool for dengue management, offering capabilities such as aerial surveillance and targeted interventions. However, its adoption in rural communities is hindered by barriers related to community engagement and acceptance. This study aims to evaluate the barriers and opportunities for community engagement in drone-based dengue management within rural Malaysian settings. A cross-sectional study was conducted across six states representing rural Malaysia: Kelantan, Terengganu, Pahang, Johor, Kedah, and Perlis. A total of 190 participants were recruited using a stratified purposive sampling method. Data were collected via structured questionnaires assessing sociodemographic characteristics, perceptions of drone technology, and willingness to engage in dengue prevention activities, such as downloading a dengue-related application or participating in mosquito control training programs. Descriptive statistics and multinomial logistic regression were used to analyze factors influencing community engagement. Participants were predominantly female (67.4%) and of Malay ethnicity (>90%). Younger participants (<40 years) showed significantly lower willingness to participate in training programs (“Maybe” vs. “No”: OR = 0.255, 95% CI: 0.067–0.968, p = 0.045), while age was not a significant predictor for app adoption. Negative perceptions of drone use and sociodemographic factors, such as housing type and residency duration, did not significantly influence willingness to engage. Despite these findings, qualitative responses highlighted concerns related to privacy, trust, and technological accessibility in rural areas. Drone-based dengue management in rural Malaysia faces challenges in community engagement, particularly among younger demographics. Tailored strategies, such as gamified training programs and targeted awareness campaigns, are necessary to address barriers and foster acceptance. These findings provide critical insights for developing inclusive and effective public health interventions leveraging drone technology in resource-limited rural settings.

## Introduction

Dengue fever, a mosquito-borne viral infection, continues to pose a major public health threat globally, particularly in tropical and subtropical regions [1,2]. *Aedes aegypti* and *Aedes albopictus*, the primary vectors of dengue, thrive in environments where stagnant water provides ideal breeding grounds [3]. Conventional vector control strategies, including chemical insecticides, larval source reduction, and public awareness campaigns, have been the cornerstone of dengue prevention [4,5]. However, the effectiveness of these interventions is increasingly compromised by challenges such as the development of insecticide resistance, environmental concerns, and inconsistent community participation [6,7]. These limitations highlight the urgent need for innovative, sustainable solutions to complement existing approaches. Drone technology has emerged as a promising innovation for dengue vector management, offering capabilities such as aerial mapping, real-time surveillance, and targeted interventions [8,9]. In urban settings, drones have demonstrated potential in identifying breeding sites, optimizing intervention strategies, and improving overall efficiency in vector control [10]. However, research on drone-based solutions has predominantly focused on urban environments, where infrastructure, resource availability, and technological adoption are more feasible. There remains a critical knowledge gap regarding the applicability, feasibility, and acceptance of drone-based interventions in rural settings, particularly in Malaysia.

Unlike urban areas, rural regions present unique challenges that complicate the implementation of public health interventions. These include dispersed populations, limited infrastructure, and distinct socio-cultural dynamics that can influence the adoption of new technologies [11]. In Malaysia, dengue is an ongoing public health crisis, with thousands of cases reported annually [12]. While urban centers are often prioritized due to higher population densities and rapid urbanization, rural areas also bear a significant burden of the disease. These areas face additional challenges in dengue control, including limited access to healthcare facilities, fragmented public health services, and logistical difficulties in implementing large-scale interventions [13–15]. Moreover, rural communities exhibit diverse socio-demographic profiles, varying levels of education, and deeply rooted cultural beliefs, which can influence their acceptance of emerging technologies such as drones [16].

Despite the potential of drones for vector control, there is limited empirical evidence on how rural communities perceive this technology, their willingness to engage with it, and the potential barriers to its acceptance and implementation. Additionally, rural Malaysia presents distinct geographic and environmental challenges, such as expansive agricultural landscapes, scattered water bodies, and lower housing densities, which create undetected breeding grounds for *Aedes* mosquitoes. However, the success of UAV-based interventions relies heavily on community engagement, as acceptance and cooperation are critical for effective implementation. Without active participation, efforts to identify and eliminate breeding sites using drones may face resistance, leading to reduced intervention efficacy. Moreover, rural communities may lack awareness of the potential benefits of drone-based interventions or harbor skepticism regarding their safety, efficacy, and ethical implications [17,18]. Establishing trust and fostering community involvement can enhance intervention uptake, ensuring that UAV-based vector control strategies are sustainable and culturally appropriate. Addressing these barriers requires an in-depth understanding of rural community dynamics and the development of targeted engagement strategies that promote acceptance, facilitate knowledge-sharing, and encourage collaboration between public health authorities and local populations.

This study focuses on identifying the barriers and opportunities for community engagement in drone-based dengue management within rural Malaysia. By evaluating community perceptions, attitudes, and willingness to participate in drone-related initiatives, this manuscript seeks to uncover critical factors influencing the feasibility of implementing this technology in rural settings. The findings aim to contribute to the development of tailored, context-specific dengue management strategies that leverage drone technology while addressing the unique needs and challenges of rural Malaysian communities. Ultimately, this research seeks to bridge the gap between technological innovation and its practical application in underserved rural areas, paving the way for more inclusive and effective dengue control efforts. This study aims to identify key barriers and facilitators influencing community engagement in drone-based dengue management within rural Malaysia. By evaluating community perceptions, attitudes, and willingness to participate in drone-related initiatives, this research examines the feasibility of implementing UAV technology in rural settings. The findings will contribute to the development of tailored, context-specific dengue management strategies that integrate drone technology while addressing the unique challenges faced by rural Malaysian communities. Ultimately, this study aims to bridge the gap between technological innovation and practical application in underserved rural areas, fostering more inclusive and effective dengue control efforts.

## Materials and methods

### Study Area

This study was conducted across six rural states in Malaysia: Kelantan, Terengganu, Pahang, Johor, Kedah, and Perlis. These states were selected based on their high dengue incidence rates, diverse community structures, and geographic features conducive to drone operations, such as open landscapes and identifiable mosquito breeding hotspots. In the Malaysian context, rural areas are defined as regions with low population density, limited access to urban infrastructure, and economies primarily based on agriculture, fishing, or small-scale industries. These areas are characterized by dispersed settlements and a strong sense of community, making them both a challenge and an opportunity for the adoption of innovative technologies such as drones. The study regions encompass a range of socio-economic profiles, including variations in housing types, residency patterns, and population mobility. The land use in these areas is a mix of residential zones, agricultural land, and forested regions, providing a dynamic setting for evaluating the integration of drone-based dengue management within the unique dynamics of rural Malaysian communities.

### Study Design

**Fig 1:**
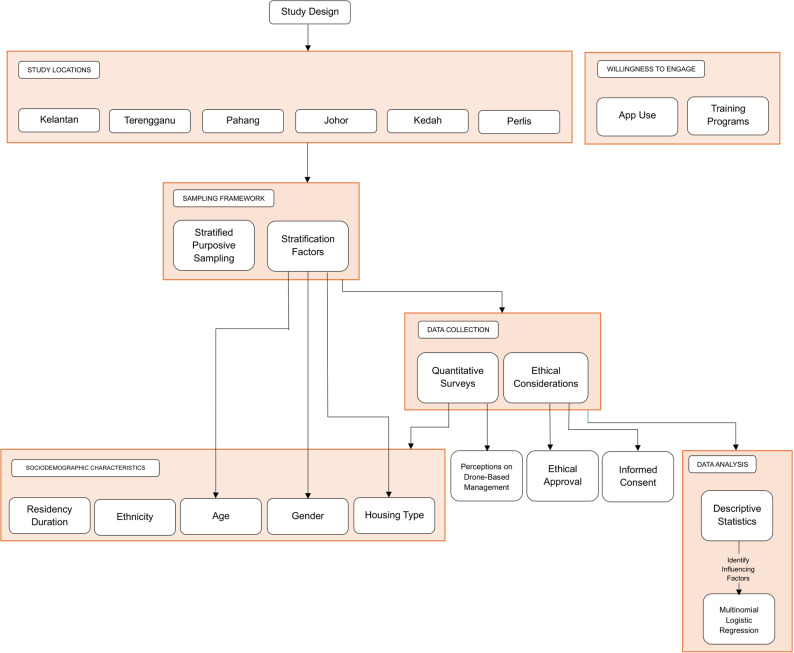
The flowchart illustrates the study design, including the six selected rural states (Kelantan, Terengganu, Pahang, Johor, Kedah, and Perlis).

This study employed a cross-sectional design to investigate barriers and opportunities for community engagement in drone-based dengue management, focusing on rural areas in Malaysia (Fig 1). A mixed-methods approach was utilized, with a primary emphasis on quantitative data collection through structured questionnaires. Additionally, qualitative insights were incorporated through open-ended survey questions (or focus group discussions/interviews, if applicable), allowing for a more comprehensive understanding of community perceptions. A total of 190 participants were recruited using a stratified purposive sampling method, ensuring representation across diverse demographic groups, including age, gender, and housing types. This approach was chosen to systematically capture variations in community perceptions and acceptance of drone technology across different rural settings. By stratifying participants based on key sociodemographic factors, the study aimed to enhance the generalizability of findings within the rural Malaysian context, accounting for differences in socioeconomic status, educational background, and geographical distribution.

The selection of six rural states was guided by their dengue burden, geographic diversity, and socio-environmental characteristics. These states were chosen to represent variations in climate, land use (e.g., agricultural, coastal, inland), and infrastructure accessibility, ensuring a more holistic understanding of how different rural communities engage with dengue control efforts. Additionally, the selected states encompass regions with differing levels of previous exposure to vector control programs, allowing for comparative insights into community readiness for drone-based interventions. The inclusion criteria required participants to be residents of the selected rural areas, aged 18 years and above, and willing to participate in the study. Data collection involved the administration of structured questionnaires to capture sociodemographic characteristics such as age, gender, ethnicity, residency duration, and housing type. Additionally, the survey assessed participants’ perceptions of drone-based dengue management and their willingness to engage in dengue prevention activities, such as downloading dengue-related applications or participating in training programs.

Ethical approval for the study was obtained from the relevant institutional review board. All participants provided written informed consent, and confidentiality and anonymity were maintained throughout the study. Participation in the study was entirely voluntary. Data analysis was conducted using descriptive and inferential statistical methods. Multinomial logistic regression was applied to identify factors influencing participants’ willingness to adopt drone-related dengue prevention measures, including app usage and participation in training programs. This methodological approach provided a robust framework for identifying key determinants of community engagement in drone-based dengue management, ensuring that the findings reflect the diverse perspectives and challenges faced by rural populations across Malaysia.

### Data collection tool and procedure

The structured questionnaire used for data collection was designed to capture participants’ sociodemographic characteristics, perceptions of drone-based dengue management, and willingness to engage in dengue prevention activities. The questionnaire used in this study was adopted from the validated instrument published by Annan et al. (2022), ensuring relevance and reliability in capturing the targeted variables [19]. It included multiple sections covering age, gender, ethnicity, residency duration, housing type, perceptions of drones for vector control, and willingness to download dengue-related applications or participate in mosquito control training programs. Participants were recruited through local community centers, public health campaigns, and outreach initiatives conducted in collaboration with local health authorities. This approach ensured representation from diverse rural populations and enhanced engagement with the study. Inclusion criteria required participants to be 18 years or older, permanent residents of the selected rural areas, and willing to provide informed consent. Exclusion criteria included individuals who had previously participated in similar studies, to avoid response bias, and those with cognitive impairments that could affect their ability to comprehend or complete the survey. Likert-scale items were used to quantify perceptions, while open-ended questions allowed participants to express their views in greater detail. To ensure clarity and reliability, the questionnaire was pretested before full deployment. The full version of the questionnaire is provided in the supplementary materials (**Supplementary File 1**) for reference.

### Variables and measurement

This study assessed key variables through structured questionnaires, categorized into three primary domains: sociodemographic characteristics, perceptions of drone-based dengue management, and willingness to engage in prevention activities. Sociodemographic variables included age, gender, ethnicity, residency duration, and housing type. These variables provided foundational insights into the participants’ demographic and residential contexts.

Perceptions of drone-based dengue management were measured using Likert-scale items (1 = strongly disagree to 5 = strongly agree) to capture attitudes toward drone use, including trust, perceived benefits, and concerns. Specific concerns such as privacy, safety, and efficacy were further explored through both closed-ended and open-ended responses. Perceived benefits of drones for dengue management were assessed using composite measures derived from questions focused on their potential to enhance vector control efforts. Willingness to engage in prevention activities was measured using categorical responses for two key indicators: willingness to download a dengue-related application and willingness to participate in mosquito control training programs (responses categorized as “yes,” “maybe,” or “no”). These variables served as key outcomes in understanding community engagement with drone-based initiatives.

The primary outcome variable was community engagement in drone-based dengue management, operationalized through willingness to adopt drone-related measures such as app usage and training participation. Multinomial logistic regression was used to analyze the associations between engagement levels and the sociodemographic or perception variables. This structured approach ensured a comprehensive evaluation of the factors influencing barriers and opportunities for implementing drone-based dengue management in rural Malaysia.

### Ethical considerations

This study was conducted in accordance with ethical guidelines and approved by the Research Ethics Committee (REC), under the reference number REC/07/2023 (ST/MR/183), issued on 21 July 2023. All participants were provided with detailed information about the study’s purpose, procedures, and their rights as participants. Written informed consent was obtained from all individuals prior to participation, ensuring that they understood the voluntary nature of their involvement. Participants were assured of the confidentiality and anonymity of their responses. No identifying information was collected or linked to the data, and all information was stored securely in password-protected systems accessible only to the research team. Additionally, participants were informed that their responses would be used solely for research purposes and that they could withdraw from the study at any time without any repercussions. This study adhered to the principles of the Declaration of Helsinki and relevant institutional and national ethical guidelines, ensuring respect, privacy, and protection for all participants involved in the research. These measures safeguarded participants’ rights and upheld the integrity of the research process.

### Data analysis and statistical methods

Data collected from the structured questionnaires were analyzed using descriptive and inferential statistical methods to explore barriers and opportunities for community engagement in drone-based dengue management. Prior to analysis, the data were reviewed for completeness and accuracy and subsequently entered into statistical software for processing. Descriptive statistics were employed to summarize participants’ sociodemographic characteristics, perceptions of drone-based dengue management, and willingness to engage in prevention activities. Categorical variables were reported as frequencies and percentages, while continuous variables were summarized as means and standard deviations.

To identify factors influencing participants’ willingness to adopt drone-based dengue prevention measures, multinomial logistic regression was conducted. The dependent variables included willingness to download a dengue-related application and willingness to participate in mosquito control training programs, each categorized as “yes,” “maybe,” or “no.” Independent variables included sociodemographic characteristics (e.g., age, gender, ethnicity, residency duration, and housing type) and perceptions of drones (e.g., attitudes, concerns, and perceived benefits). The strength and direction of associations were measured using odds ratios (ORs) with 95% confidence intervals (CIs). Statistical significance was set at a p-value threshold of <0.05.

Additionally, open-ended responses were analyzed using thematic analysis to identify key themes and insights related to participants’ perceptions and concerns about drone-based dengue management. These qualitative findings complemented the quantitative results, providing a deeper understanding of the contextual challenges and opportunities for implementing drone-based strategies in rural Malaysia. This comprehensive analytical approach ensured robust and nuanced insights into the study objectives.

### Operational definitions

Key terms and concepts in this study were operationally defined to ensure consistency in interpretation and analysis. Community engagement was defined as the active participation of individuals and groups within rural communities in drone-based dengue management initiatives, measured by their willingness to engage in activities such as downloading dengue-related applications or attending mosquito control training programs [20]. Drone-based dengue management referred to the use of drone technology for surveillance and control of dengue vector breeding sites, including mapping, monitoring, and targeted interventions to reduce mosquito populations and mitigate dengue transmission [21].

Rural areas were defined according to the Malaysian rural classification as regions with low population density, limited urban infrastructure, and economies primarily reliant on agriculture, fishing, or small-scale industries [22]. Participants’ willingness to download dengue-related applications was assessed as their intention to use a mobile application linked to drone-based dengue management, categorized into “yes,” “maybe,” or “no.” Similarly, willingness to participate in mosquito control training was measured as their intention to attend training sessions on mosquito control techniques within the context of drone-based initiatives, also categorized into “yes,” “maybe,” or “no.”

Perceptions of drones encompassed participants’ attitudes, concerns, and perceived benefits regarding the use of drones for dengue management, evaluated using Likert-scale items and thematic analysis of open-ended responses. Sociodemographic characteristics included participants’ age, gender, ethnicity, residency duration, and housing type, providing a context for analyzing their engagement with drone-based dengue management strategies. These operational definitions ensured clarity and alignment throughout the study, facilitating robust and reproducible analysis.

## Results

### Sociodemographic characteristics

The study analyzed responses from 190 participants across six rural Malaysian states: Kelantan, Terengganu, Pahang, Johor, Kedah, and Perlis as summarized in **Table 1**. The gender distribution skewed heavily towards females (67.4%), with notable disparities in some states, such as Johor (87.5% female) and Pahang (80% female). In contrast, Terengganu presented a more balanced gender distribution (53.5% male, 46.5% female). Age distribution patterns varied by state, with Kelantan and Kedah showing a relatively younger demographic (30.9% and 33.3%, respectively, aged 18–30 years). Pahang and Johor, however, had the highest proportion of respondents aged 31–40 years (68% and 50%, respectively). Perlis stood out, with 50% of respondents in the 41–50 age group, reflecting a significantly older population compared to other states. The ethnic composition was predominantly Malay, exceeding 90% across all states, consistent with the rural demographic profile of Malaysia. Residency duration varied, with Perlis and Kedah having the highest proportion of long-term residents (50% and 43.3%, respectively, residing >3 years), while Kelantan showed higher mobility, with 22.1% living in the area for less than 6 months. Housing types were diverse, with single-family houses dominating in Perlis (75%) and Kedah (50%), while terrace houses were more prevalent in Johor (50%) and Pahang (40%). These findings provide a comprehensive overview of the sociodemographic landscape of rural Malaysia, which forms the basis for analyzing community attitudes towards drone-based dengue management.

**Table 1:** Sociodemographic characteristics of respondents from six rural states in Malaysia, highlighting gender, age distribution, ethnicity, residency duration, housing type, and household size.

		States, n (%)					
Characteristics		Kelantan	Terengganu	Pahang	Johor	Kedah	Perlis
**Gender**	Male	22 (32.4)	23 (53.5)	5 (20.0)	2 (12.5)	8 (26.7)	4 (50.0)
	Female	46 (67.6)	20 (46.5)	20 (80.0)	14 (87.5)	22 (73.3)	4 (50.0)
**Age Group**	18-30 years old	21 (30.9)	7 (16.3)	7 (28.0)	7 (43.8)	10 (33.3)	2 (25)
	31-40 years old	14 (20.6)	17 (39.5)	17 68.0)	8 (50.0)	9 (3)	1 (12.5)
	41-50 years old	16 (23.5)	12 (27.9)	1 (4.0)	1 (6.2)	7 (23.3)	4 (50)
	51-60 years old	11 (16.2)	1 (2.3)	0 (0.0)	0 (0.0)	1 (3.3)	0 (0.0)
	>61 years old	6 (8.8)	6 (14.0)	0 (0.0)	0 (0.0)	3 (1)	1 (12.5)
**Race**	Malay	67 (98.5)	39 (90.6)	24 (96.0)	16 (100.0)	30 (100.0)	8 (100.0)
	Chinese	1 (1.5)	2 (4.7)	1 (4.0)	0 (0.0)	0 (0.0)	0 (0.0)
	Indian	0 (0.0)	2 (4.7)	0 (0.0)	0 (0.0)	0 (0.0)	0 (0.0)
	Others	0 (0.0)	0 (0.0)	0 (0.0)	0 (0.0)	0 (0.0)	0 (0.0)
**Duration of living in the area**	Below 6 months	15 (22.1)	4 (9.3)	4 (16.0)	2 (12.5)	2 (12.5)	0 (0.0)
	6 months to 1 year	16 (23.5)	5 (11.6)	9 (36.0)	9 (56.2)	10 (6)	2 (25.0)
	1 to 3 years	28 (41.2)	14 (32.6)	7 (28.0)	3 (18.8)	12 (40.0)	2 (25.0)
	Above 3 years	9 (13.2)	20 (46.5)	5 (20.0)	2 (12.5)	6 (20.0)	4 (50.0)
**Type of House**	Bungalow	13 (19.1)	9 (20.9)	4 (16.0)	1 (6.2)	5 (16.7)	2 (25.0)
	Semi-Detached	15 (22.1)	9 (20.9)	4 (16.0)	3 (18.8)	5 (16.7)	0 (0.0)
	Terrace	11 (16.1)	8 (18.6)	10 (40.0)	8 (50.0)	5 (16.7)	0 (0.0)
	Townhouse	8 (11.8)	6 (14.0)	0 (0.0)	0 (0.0)	0 (0.0)	0 (0.0)
	Flat/Apartment	0 (0.0)	0 (0.0)	0 (0.0)	0 (0.0)	0 (0.0)	0 (0.0)
	Single family house	21 (30.9)	11 (25.6)	7 (28.0)	4 (25.0)	15 (50.0)	6 (75.0)
**Number of Households**	1	7 (10.3)	4 (9.3)	1 (4.0)	1 (6.2)	1 (3.3)	0 (0.0)
	2	23 (33.9)	5 (11.6)	8 (32.0)	5 (31.3)	3 (10.0)	2 (25.0)
	3	17 (25.0)	10 (23.3)	4 (16.0)	2 (12.5)	14 (46.7)	0 (0.0)
	4	12 (17.6)	16 (37.2)	9 (36.0)	7 (43.8)	9 (30.0)	3 (37.5)
	5 or more	9 (13.2)	8 (18.6)	3 (12.0)	1 (6.2)	3 (10.0)	3 (37.5)
**Total (n)**		**68**	**43**	**25**	**16**	**30**	**8**

### Community Preparedness in Using Drones for Dengue Management

***Willingness to Download a Drone-Related App***. The univariate multinomial regression analysis did not identify any significant predictors for willingness to download a drone-related dengue application. Gender, age, and housing type showed no meaningful associations, with p-values consistently exceeding 0.05. For instance, the odds ratio (OR) for male versus female participants regarding “Maybe” willingness was 1.028 (95% CI: 0.276–3.822, p = 0.967), and for “Yes” willingness, it was 1.228 (95% CI: 0.469–3.215, p = 0.676), indicating negligible effects of gender on app adoption. Similarly, negative perceptions and concerns about drones did not significantly influence app-related willingness. For example, participants who had no concerns about drones were 1.935 times more likely to express willingness to download the app compared to those with concerns (95% CI: 0.731–5.127, p = 0.184); however, the wide confidence interval suggests variability and limits the reliability of this effect. These findings imply that sociodemographic factors and individual attitudes toward drones are not primary determinants of app adoption in rural communities. Instead, other structural barriers—such as internet accessibility, digital literacy, and smartphone availability—may play a more critical role in shaping willingness to engage with technology-based vector control solutions (Table 2A).

**Table 2:** Multinomial Regression Analysis of Factors Influencing Willingness to Download a Dengue Application and Participate in Dengue Prevention Training Programs.

Independent variables	Download app (0=No, 1=Yes, 2=Maybe)	A. Willingness to download dengue application			B. Willingness to be trained for dengue prevention		
		p-value	OR	95% CI	p-value	OR	95% CI
**Gender**							
Male vs Female	Maybe vs No	1.028	0.276, 3.822	0.967	1.333	0.366, 4.854	0.663
	Yes vs No	1.228	0.469, 3.215	0.676	1.192	0.440, 3.231	0.730
**Age**							
<40 years vs> 40 years	Maybe vs No	0.781	0.206, 2.960	0.716	0.255	0.067, 0.968	0.045
	Yes vs No	1.047	0.397, 2.760	0.926	0.617	0.227, 1.674	0.343
**Duration of living in the area**							
> 3 years vs < 3 years	Maybe vs No	0.628	0.181, 2.174	0.462	0.468	0.122, 1.803	0.270
	Yes vs No	1.494	0.575, 3.880	0.409	0.726	0.251, 2.095	0.553
**Type of house**							
Terrace vs Others	Maybe vs No	1.328	0.364, 4.851	0.667	1.356	0.356, 5.160	0.655
	Yes vs No	1.768	0.680, 4.597	0.242	2.185	0.791, 6.030	0.131
**Negative perceptions about drone use**							
No vs Unsure	Maybe vs No	1.231	0.238, 6.374	0.805	0.696	0.146, 3.313	0.649
	Yes vs No	0.821	0.251, 2.687	0.745	0.523	0.163, 1.675	0.275
Yes vs Unsure	Maybe vs No	0.793	0.166, 3.795	0.771	0.449	0.085, 2.359	0.344
	Yes vs No	0.764	0.243, 2.403	0.645	0.607	0.175, 2.107	0.432
**Concerns about drone use**							
No vs Unsure	Maybe vs No	0.745	0.147, 3.777	0.772	0.277	0.058, 1.329	0.109
	Yes vs No	1.419	0.461, 4.373	0.542	0.372	0.112, 1.237	0.107
Yes vs Unsure	Maybe vs No	1.843	0.491, 6.924	0.365	0.865	0.205, 3.639	0.843
	Yes vs No	1.935	0.731, 5.127	0.184	1.099	0.363, 3.327	0.867

**Note:** Multinomial regression analysis of factors influencing the willingness to (A) download dengue application and (B) trained for dengue prevention. The table presents odds ratios (OR), 95% confidence intervals (CI), and *p*-values for key sociodemographic variables, housing type, and perceptions regarding drone use, categorized by responses (“No,” “Maybe,” and “Yes”). Significant associations are highlighted to identify potential predictors of app adoption.

***Willingness to Participate in Mosquito Control Training:*** The analysis revealed one significant association: younger participants (<40 years) were significantly less likely to express “Maybe” willingness to participate in training compared to older individuals (OR = 0.255, 95% CI: 0.067–0.968, p = 0.045). This finding highlights a potential engagement gap among younger demographics, suggesting that traditional training formats may not effectively reach this age group, which could be due to competing economic priorities, lower perceived personal risk, or limited awareness of training benefits. However, no significant differences were observed for the “Yes” versus “No” comparison (p > 0.05), indicating that younger participants were not outright rejecting training opportunities but were more hesitant than their older counterparts. Other demographic and perceptual factors including gender (OR for “Yes” vs. “No” = 1.192, 95% CI: 0.440–3.231, p = 0.730), residency duration (OR for “Yes” vs. “No” = 0.726, 95% CI: 0.251–2.095, p = 0.553), and housing type (OR for “Yes” vs. “No” = 2.185, 95% CI: 0.791–6.030, p = 0.131) did not show significant associations with willingness to participate in mosquito control training. Additionally, negative perceptions and concerns about drones exhibited negligible effects, with wide confidence intervals and p-values exceeding 0.05 (Table 2B).

The findings suggest that targeted engagement strategies are necessary to encourage app adoption and training participation in rural communities. Since demographic and perceptual factors were not significant predictors, policymakers should focus on addressing external barriers, such as improving internet access, increasing technology literacy, and tailoring community-based training programs to be more accessible and engaging for younger populations. Approaches such as gamified training models, mobile-based learning, or financial incentives could enhance participation among younger demographics. Additionally, future studies should consider a larger sample size or mixed-method approaches to further investigate the nuanced factors influencing community engagement in drone-based dengue control strategies.

**Fig 2:**
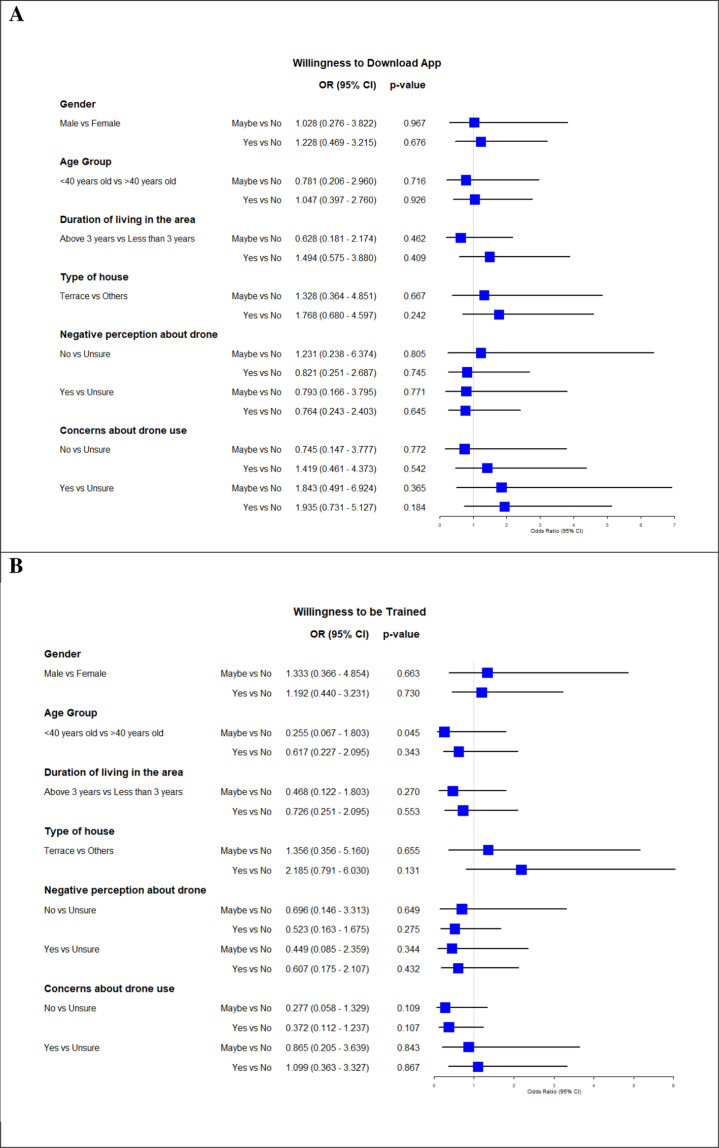
Forest plots illustrating factors influencing (A) willingness to download a dengue prevention application and (B) willingness to be trained for dengue prevention. Odds ratios (OR) with 95% confidence intervals (CI) are presented for key sociodemographic variables, housing type, and perceptions regarding drone use, highlighting significant and non-significant predictors for each outcome.

***Comparative Analysis of Key Factors***: The forest plots (Fig 2) provide a comparative visualization of the odds ratios (ORs) and confidence intervals (CIs) for factors influencing willingness to adopt drone-based dengue management strategies. These plots summarize key predictors across two primary outcomes: (A) willingness to download a dengue prevention application and (B) willingness to participate in mosquito control training.

For willingness to download a drone-related dengue app, the forest plot (Fig 2A) indicates that gender, residency duration, and housing type had negligible effects, as their ORs were close to 1, with wide confidence intervals crossing the null value. This suggests that these sociodemographic factors do not significantly influence app adoption. Similarly, negative perceptions and concerns about drones did not emerge as significant barriers, reinforcing that community engagement with the app is not strongly influenced by attitudes toward drone technology. Instead, other external factors such as internet access, digital literacy, or previous exposure to technology-driven public health initiatives may play a more significant role in shaping app adoption patterns. For willingness to participate in mosquito control training, the forest plot (Fig 2B) highlights one statistically significant finding: younger individuals (<40 years) were significantly less likely to express “Maybe” willingness to participate in training compared to older individuals (OR = 0.255, 95% CI: 0.067–0.968, p = 0.045). This suggests that younger demographics may require tailored engagement strategies to encourage participation in structured training programs. Their lower willingness could stem from competing economic or personal priorities, a lower perceived risk of dengue, or a lack of interest in community-led vector control initiatives. No other demographic variables, including gender, residency duration, and housing type, showed significant associations with training participation, reinforcing the need for alternative strategies to engage younger populations.

The findings from the forest plots emphasize that sociodemographic factors and perceptions about drones do not serve as major barriers to community preparedness for drone-based dengue management. However, the observed reluctance among younger participants to engage in training highlights a critical area for intervention. Tailored strategies, such as gamified training modules, financial incentives, and mobile-based learning approaches, may be necessary to increase participation in mosquito control programs among younger demographics. Additionally, future research should explore unmeasured external factors, such as technological accessibility, digital literacy, and prior experience with health-related mobile applications, to gain deeper insights into the determinants of app adoption and training engagement. These insights will be crucial for developing policies that ensure equitable access to drone-based dengue control strategies across diverse rural communities.

## Discussion

The findings of this study highlight the unique challenges and opportunities associated with implementing drone-based initiatives for dengue management in rural communities. Rural areas often face significant barriers to healthcare interventions, including limited access to infrastructure, educational resources, and technology [23]. Despite these challenges, the acceptance of mobile applications for vector surveillance among participants suggests a readiness to adopt technological solutions, even in resource-limited settings [24]. This finding underscores the potential of technology, such as drones and mobile apps, to bridge existing gaps in public health systems in rural regions.

Rural areas, particularly in Malaysia, are characterized by distinct sociodemographic and geographic factors that influence community participation in health interventions. For instance, the study revealed that younger participants (<40 years) in rural areas were less willing to participate in mosquito control training programs. This demographic often faces competing priorities, such as economic pressures or limited awareness of the importance of such initiatives. The lower engagement among younger populations highlights the need for policy-driven approaches to incentivize participation, such as incorporating mosquito control education into school curriculums or workforce training programs. Additionally, government or local health authorities could introduce subsidies, rewards, or certification programs to encourage participation in dengue prevention initiatives. Flexible, accessible, and incentivized training methods such as gamified learning or mobile-based training sessions could further address the engagement gap among younger demographics.

Building on previous research, such as Erku et al. (2023), this study underscores the relevance of digital tools in overcoming rural healthcare challenges. Erku et al. demonstrated that mobile apps and drones can enhance community engagement in under-resourced areas by providing efficient, scalable, and user-friendly solutions [25]. However, the successful integration of these tools into public health strategies requires targeted policies that address existing technological disparities. For example, government-led initiatives could focus on providing subsidies for mobile data plans in rural areas, ensuring that mobile health (mHealth) applications are accessible to all socioeconomic groups. Additionally, policymakers should consider investing in community-based technology literacy programs to empower rural populations with the necessary skills to effectively engage with drone and app-based interventions.

In rural Malaysia, where logistical and manpower constraints hinder traditional dengue control measures, the use of drones for vector surveillance and mobile apps for real-time alerts represents a transformative approach to addressing these limitations. However, the success of these interventions depends on community acceptance, technological accessibility, and trust in new technologies. The study suggests that drones face higher resistance compared to mobile apps, primarily due to cost, accessibility, and lack of familiarity. Drones require specialized infrastructure, trained personnel, and regulatory approval, making them more complex to adopt than mobile applications, which are already widely available on personal devices. Additionally, privacy concerns and data security issues were identified as key barriers to drone adoption, indicating a need for clear regulatory frameworks to build public trust in drone-based interventions. National vector control policies should include guidelines on ethical drone usage, data protection, and community consent processes to ensure transparency and responsible deployment. Rural-specific concerns about drone usage, including cultural perceptions, skepticism, and fear of surveillance, may also contribute to resistance. While no significant associations were found between perceptions of drones and willingness to participate, these issues can still influence the long-term acceptance and scalability of drone-based interventions. In contrast, mobile apps may be perceived as more user-friendly and less intrusive, leading to higher acceptance rates. To enhance community engagement with drone technology, public health authorities should implement participatory approaches where rural communities are actively involved in decision-making, planning, and implementation. Strategies such as community demonstrations, pilot programs, and interactive workshops can help familiarize residents with drone technology, dispel misconceptions, and increase trust in its application. Additionally, subsidies or financial incentives for drone-based initiatives could help alleviate concerns about cost and accessibility, further promoting community buy-in.

Additionally, the challenges of sustained engagement in rural settings are well-documented in the literature. Strasser et al. (2016) highlighted that rural populations often lack consistent access to educational and public health resources, which limits their participation in health programs [26]. This is consistent with the findings of this study, where the duration of residency, housing type, and other sociodemographic factors were not significant predictors of willingness to engage in training or adopt mobile apps. These results suggest that, in rural settings, external factors such as the availability of community resources, local leadership, and culturally relevant approaches may play a more critical role in determining the success of public health initiatives [27]. This underscores the need for intersectoral collaboration, where local government agencies, non-governmental organizations (NGOs), and community-based organizations (CBOs) work together to implement and sustain vector control initiatives. Establishing partnerships with community leaders and influencers could enhance engagement and ensure interventions are culturally and contextually appropriate [28].

Despite these challenges, the findings support the potential of drone-based interventions to enhance rural healthcare outcomes [29]. However, the effectiveness of such solutions depends on addressing the systemic inequalities faced by rural populations, such as limited internet connectivity, technological literacy, and access to funding for public health programs. Policy recommendations should focus on integrating these technologies into existing rural healthcare infrastructures, ensuring they are accessible and sustainable in the long term. National and state-level health authorities should consider incorporating drone-based surveillance into broader dengue control policies, securing dedicated funding for the maintenance and expansion of these programs. By embedding drone-based vector control strategies within Malaysia’s national dengue prevention framework, policymakers can ensure that these innovations are not only effective but also scalable and inclusive.

## Strengths, limitations, and recommendations for future research

This study presents several strengths, making it a significant contribution to public health research in rural Malaysia settings. It highlights the innovative use of drone-based technology and mobile applications for dengue management an underexplored area in resource-limited settings. By including diverse rural states, the research captures a broad range of sociodemographic and geographic contexts, offering a comprehensive understanding of community dynamics. Additionally, the methodological rigor, particularly the use of multinomial regression analysis, provides valuable insights into factors influencing community preparednes**s**. The dual focus on app adoption and mosquito control training programs enhances the study’s relevance by offering a balanced perspective on technological and behavioral components. Furthermore, the findings have practical implications, identifying demographic gaps, such as lower engagement among younger populations, and offering evidence-based recommendations for public health practitioners.

Despite these contributions, the study has several limitations that should be addressed in future research. The focus on six rural states and a sample size of 190 respondents limits the generalizability of findings to other regions or communities with differing socio-economic contexts. To improve external validity, future studies should aim for a sample size of at least 400–600 participants, depending on population diversity and regional representation. A larger sample would allow for more robust statistical analyses, greater subgroup comparisons, and improved reliability of findings across different demographic and geographic settings. Additionally, the reliance on self-reported data introduces potential biases, such as social desirability bias or recall inaccuracies, which could affect the validity of responses. The absence of qualitative methods restricts a deeper exploration of community perceptions and concerns, limiting the study’s ability to capture nuanced attitudes toward drone-based interventions. Future studies should integrate focus groups, in-depth interviews, or participatory action research to provide richer contextual insights into barriers and enablers of community engagement. Furthermore, this study primarily emphasizes sociodemographic factors, overlooking other critical determinants such as trust in authorities, prior exposure to technology, and infrastructural constraints that could significantly influence community acceptance of UAV-based dengue control measures. Short data collection periods and a lack of investigation into technological and infrastructural challenges, including internet connectivity and drone operational feasibility, further limit the comprehensiveness of the findings. Future research should adopt longitudinal designs to assess the sustained impact of drone interventions, evaluate cost-effectiveness, and investigate the role of public-private partnerships in scaling up UAV-based vector control strategies.

To enhance the successful adoption and implementation of drone-based dengue management, several policy and intervention strategies should be considered. First, integrating drone-based surveillance into national dengue control programs is essential. Establishing formal guidelines and protocols for drone use in vector surveillance, fostering collaborations between government agencies, local health authorities, and private technology providers, and securing sustainable funding will ensure the long-term viability of these initiatives. Additionally, improving digital literacy and community readiness is crucial for fostering public trust and engagement. Implementing technology training workshops in rural areas, developing educational campaigns to address privacy concerns and ethical considerations, and involving local community leaders and health volunteers as intermediaries can facilitate greater acceptance of drone-based vector control measures.

Another critical aspect is the need for targeted engagement strategies for younger populations, as findings indicate lower willingness to participate in mosquito control training among individuals under 40 years old. Strategies such as gamified training modules, incentives like certification or financial rewards, and the inclusion of dengue control education in school curricula and vocational training programs can encourage youth participation. Furthermore, enhancing the usability and accessibility of mobile applications will improve adoption rates. Optimizing mobile app design to ensure compatibility with low-cost smartphones, developing multi-language support with culturally relevant content, and exploring government subsidies or incentives for smartphone access in low-income communities can significantly increase engagement.

Beyond community engagement, addressing technological and infrastructure barriers is imperative for ensuring equitable access to drone-based interventions. Expanding rural internet connectivity will support real-time data collection and drone operations, while feasibility studies on cost-effective drone deployment models will ensure sustainability and scalability. Additionally, the development of a structured maintenance and monitoring framework for drones used in public health programs will be necessary to ensure their continued effectiveness. By implementing these targeted policies and interventions, stakeholders can maximize the potential of drone-assisted dengue control while ensuring that these technological solutions are accessible, sustainable, and responsive to the unique challenges of rural communities.

## Conclusion

In conclusion, this study demonstrates the potential of drone-based dengue management in rural Malaysia, highlighting the readiness of rural communities to adopt mobile applications for vector surveillance. However, it also underscores the need for targeted interventions to address demographic gaps, such as the lower engagement among younger participants, and to adapt technological solutions to the unique challenges of rural settings. To maximize the real-world impact, policymakers and public health practitioners should integrate drone-based surveillance into national dengue control frameworks, ensuring that regulatory guidelines support ethical drone deployment, data security, and community participation. Public health agencies should develop targeted awareness campaigns, training programs, and incentives to increase engagement, particularly among younger populations. Additionally, investments in rural digital infrastructure and mobile health technologies will be crucial for enhancing accessibility and sustainability. By combining innovative tools with community-driven approaches and addressing rural-specific barriers, policymakers and health practitioners can optimize resource allocation, improve disease surveillance efficiency, and enhance the sustainability of dengue control programs in underserved areas.
